# Feasibility of mapping and ablating ectopy-triggering ganglionated plexus reproducibly in persistent atrial fibrillation

**DOI:** 10.1007/s10840-023-01517-9

**Published:** 2023-03-03

**Authors:** Clare Coyle, Simos Koutsoftidis, Min-Young Kim, Bradley Porter, Daniel Keene, Vishal Luther, Balvinder Handa, Jamie Kay, Elaine Lim, Louisa Malcolme-Lawes, Michael Koa-Wing, Phang Boon Lim, Zachary I. Whinnett, Fu Siong Ng, Norman Qureshi, Nicholas S. Peters, Nicholas W. F. Linton, Emmanuel Drakakis, Prapa Kanagaratnam

**Affiliations:** 1 NHLI, Imperial College London, London, UK; 2 Imperial Centre for Cardiac Engineering, Imperial College London, London, UK; 3 Department of Cardiology, Hammersmith Hospital, Du Cane Road, London, W12 0HS UK; 4 Department of Bioengineering, Imperial College London, London, UK

**Keywords:** Atrial fibrillation, Autonomic, Ganglionated plexus, High-frequency simulation, Ablation

## Abstract

**Background:**

Ablation of autonomic ectopy-triggering ganglionated plexuses (ET-GP) has been used to treat paroxysmal atrial fibrillation (AF). It is not known if ET-GP localisation is reproducible between different stimulators or whether ET-GP can be mapped and ablated in persistent AF. We tested the reproducibility of the left atrial ET-GP location using different high-frequency high-output stimulators in AF. In addition, we tested the feasibility of identifying ET-GP locations in persistent atrial fibrillation.

**Methods:**

Nine patients undergoing clinically-indicated paroxysmal AF ablation received pacing-synchronised high-frequency stimulation (HFS), delivered in SR during the left atrial refractory period, to compare ET-GP localisation between a custom-built current-controlled stimulator (Tau20) and a voltage-controlled stimulator (Grass S88, SIU5). Two patients with persistent AF underwent cardioversion, left atrial ET-GP mapping with the Tau20 and ablation (Precision™, Tacticath™ [*n* = 1] or Carto™, SmartTouch™ [*n* = 1]). Pulmonary vein isolation (PVI) was not performed. Efficacy of ablation at ET-GP sites alone without PVI was assessed at 1 year.

**Results:**

The mean output to identify ET-GP was 34 mA (*n* = 5). Reproducibility of response to synchronised HFS was 100% (Tau20 vs Grass S88; [*n* = 16] [kappa = 1, SE = 0.00, 95% CI 1 to 1)][Tau20 v Tau20; [*n* = 13] [kappa = 1, SE = 0, 95% CI 1 to 1]). Two patients with persistent AF had 10 and 7 ET-GP sites identified requiring 6 and 3 min of radiofrequency ablation respectively to abolish ET-GP response. Both patients were free from AF for > 365 days without anti-arrhythmics.

**Conclusions:**

ET-GP sites are identified at the same location by different stimulators. ET-GP ablation alone was able to prevent AF recurrence in persistent AF, and further studies would be warranted.

## Introduction

Atrial fibrillation (AF) is triggered by ectopic beats from the pulmonary veins (PVs) [1]. Several studies have shown that such PV ectopy can be generated by stimulating the left atrial intrinsic cardiac autonomic nervous system [2–6]. These studies imply that the atrial neural network is important in the pathogenesis of AF. Analysis of the histology of this network in post-mortem human hearts demonstrated dense clusters of nerves in the atrial epicardium called ganglionated plexuses (GP) [2]. These sites could be targeted for ablation by an anatomical approach alone. However, this would be more difficult to control between operators and varying atrial anatomy. The use of high-frequency stimulation (HFS) to identify GP sites is reproducible and gives a clear endpoint for ablation. The first functional studies of this network in patients identified GP sites exhibiting atrioventricular dissociation (AVD) in response to several seconds of continuous high-frequency stimulation (HFS) [3–6]. Subsequently, ectopy-triggering (ET) sites were also located by a different method delivering short bursts of HFS during the atrial refractory period and observing for a triggered ectopic response [7, 8]. Global left atrial GP mapping has been used to create anatomical maps of the locations of GPs and whilst there is anatomical overlap of AVD and ET GPs in a proportion of cases this is not complete [9]. This has encouraged the use of separate HFS mapping approaches. ET-GP can only be located in sinus rhythm, and AF induced by ET-GP stimulation can sometimes become resistant to cardioversion precluding further ET-GP mapping. Consequently, it has been assumed that ET-GP mapping would be futile during persistent AF and the distribution of ET-GP in patients with persistent AF is not known.

The GANGLIA-AF trial showed that when the entire atrial ET-GP could be mapped and ablated in patients with paroxysmal AF, freedom from atrial arrhythmias was similar to a standard pulmonary vein isolation procedure [8, 10, 11]. The trial used the Grass S88 stimulator which is not licenced for routine clinical work but has been used in a wide variety of autonomic research studies. It is not known whether the localisation of ET-GP sites is in any way stimulator specific. We designed and built a custom stimulator (Tau20), meeting IEC 60,601–1 3rd Edition Regulatory Standards, with similar HFS delivery profile to the Grass stimulator. Following bench and animal testing, approval was granted for a comparative study of the efficacy and reproducibility of this current-controlled stimulator (Tau20) against the voltage-controlled Grass stimulator with SIU5 in patients undergoing paroxysmal AF ablation. Feasibility of mapping ET-GP in persistent AF was also tested using the Tau20 stimulator in order to justify a larger study looking at identification and ablation of ET-GP sites in persistent AF.

## Methods

Patients undergoing clinically indicated AF ablation consented to participate. All cases were performed under general anaesthetic. Patients were excluded if they were unable to provide informed consent, developed a contraindication to catheter ablation (e.g. presence of cardiac thrombus), valvular disease moderate or greater or EF < 30%. Reproducibility of response was analysed using Cohen’s kappa test at the location level to assess within patient agreement. The research was approved by the NHS Research Ethics Committee (REC) London-Fulham Ref:14/LO/2044 and 17/LO/0465, and the South central-Berkshire 20/SC/0081.

### The Grass S88 stimulator

The Grass S88 stimulator has been widely used for autonomic studies. It has two primary output channels (S1 and S2). The S1 outputs stimuli to pace the atrium and S2 delivers short and repeated bursts of (HFS) synchronised to the paced stimuli from S1. Each channel is connected to an isolation unit (SIU5), which converts the Grass output to isolated constant voltage.

### The Tau20

**Fig. 1:**
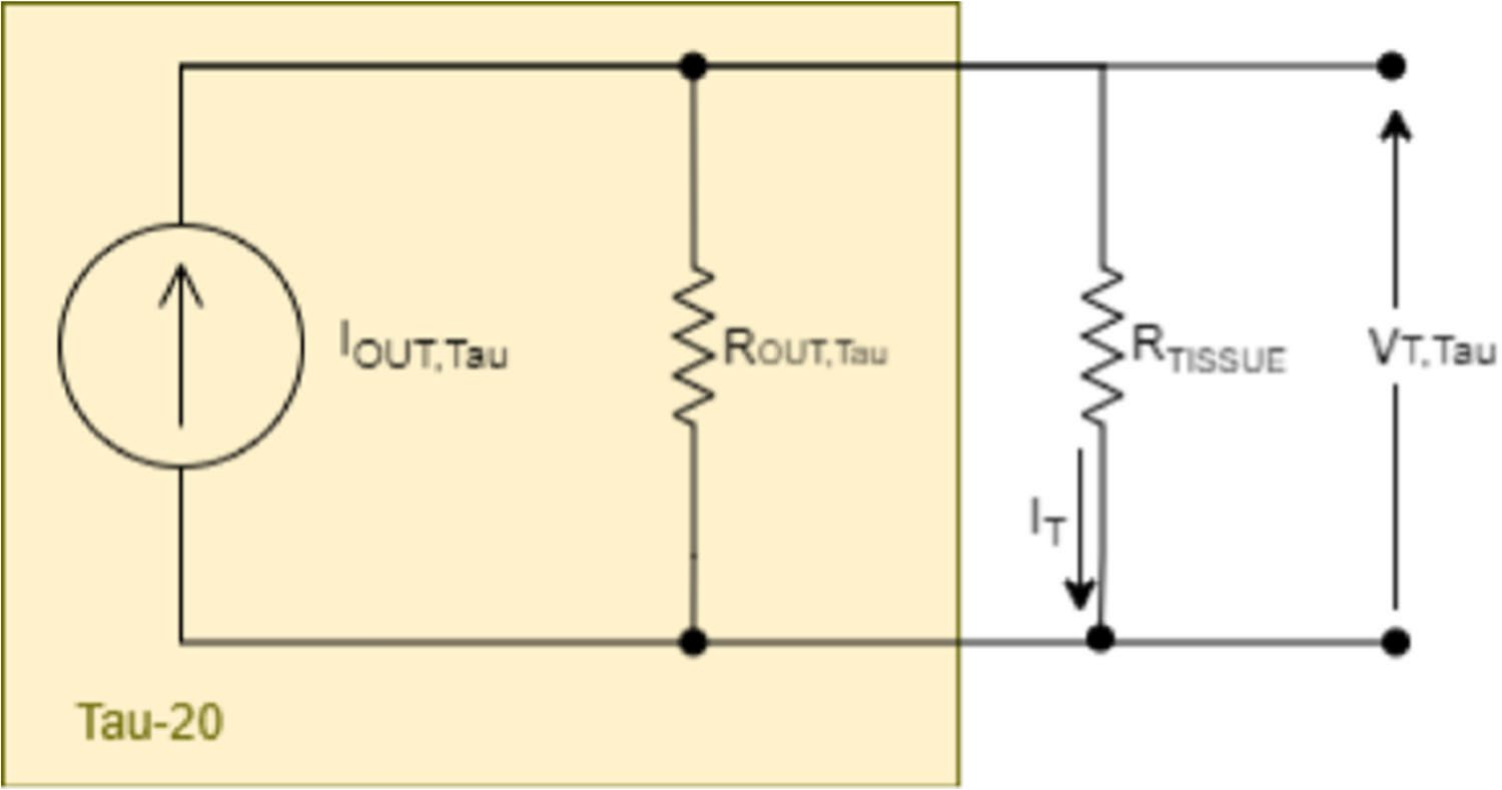
A simplified model of the Tau20 setup. The output impedance (R_OUT,TAU_, > 300 KΩ by specification) of the Tau20 is much higher than the tissue impedance (R_TISSUE_ ~ 0.2 KΩ based on Langendorff observations) resulting in the stimulating current (I_TAU,OUT_) being delivered practically in totality to the tissue. Internal Tau20 circuitry prevents the output voltage (V_T,TAU_) developed across the tissue (typically during HFS stimulation, V_T,TAU_ is the differential voltage across MAP1–2) from exceeding 15 V

The Tau20 is a current-controlled high-frequency stimulator. Figure 1 illustrates the simplified model of Tau20 when delivering constant amplitude current pulses to the tissue (range 1–100 mA).

### Identification of ET-GP

ET-GP can only be identified during sinus rhythm. HFS was delivered to the left atrial endocardium via a standard electrode catheter (Navistar™ and Tacticath™), synchronised to a paced train to deliver HFS within the atrial refractory period (80–160-ms duration at 40 Hz, 10 V, up to 15 trains). This ensures ET-GP (not atrial tissue) are specifically recruited. A positive response was atrial or PV ectopy and/or AF [9, 10, 12, 13]. Test sites were marked on the left atrial geometry created using commercially available electroanatomic mapping systems (CARTO™ & Precision™). Previous studies [8, 9] have indicated that complete GP mapping of the left atrium prior to therapeutic ablation adds roughly 60 min to the total procedure time. We elected not to perform mapping of the whole left atrium in these validation cases.

### Threshold testing for ET-GP response using Tau20

ET-GP mapping was performed using the Tau20 at an HFS output of 50 mA. The threshold for this response was determined by reducing the output by 10 mA increments until the effect was lost. The threshold at which the response was regained was assessed by increasing the output from 10 mA.

### Reproducibility of ET-GP identification by Tau20

A left atrial ET-GP map was created using the Tau20 at an output of 50 mA and then repeating HFS at the same sites with the same parameters to see if the same functional response was identified. Reproducibility of the Grass stimulator against itself has previously been validated [6, 9, 12].

### Reproducibility of ET-GP identification between Tau20 and Grass stimulator

A left atrial ET-GP map was created with one stimulator and the same locations tested with the alternative stimulator (Tau20 and Grass S88 with SIU5) to assess reproducibility. Tau20 output was 50 mA and the Grass S88 output 100 V (equivalent to ~ 10 V at the tissue). Reproducibility of both positive and negative responses was calculated.

### Feasibility testing of ET-GP mapping and ablation in persistent AF

Two patients undergoing ablation for persistent AF were recruited. A summary of the demographics of these patients can be seen in Table 1. The procedure was performed under general anaesthetic with interrupted anticoagulation and trans-oesophageal echo to confirm no left atrial appendage thrombus. Direct current external cardioversion with concomitant amiodarone use was planned to maintain sinus rhythm. The Tau20 was used to map ET-GP with synchronised HFS via the Navistar™ or Tacticath™ catheters. ET-GP sites were ablated immediately and retested to ensure loss of response. Anatomical test locations were marked on the 3D anatomy using CARTO™ or Precision™. No PVI was performed. The patient was followed up with clinic visits and Holters for 1 year to assess recurrence of AF.

**Table 1:** Demographics of study participants

Characteristic	Paroxysmal *n* = 9	Persistent *n* = 2
Age (y)	63 (± 10)	77 (± 0)
Female	2 (22%)	0 (0%)
BMI (kg/m^2^)	27.5 (± 2)	25(± 3)
Duration of AF (days)	NA	1583 (± 540)
CHA_2_DS_2_Vasc score		
0	2 (22%)	0 (0%)
1	4 (44%)	0 (0%)
2	1 (11%)	1 (50%)
≥ 3	2 (44%)	1 (50%)
Co-morbidities:		
Stroke/TIA	1 (11%)	0 (0%)
Coronary artery disease	1 (11%)	1 (50%)
Hypertension	3 (33%)	1 (50%)
Diabetes mellitus	0 (0%)	0 (0%)
Left atrial diameter (mm)	43 (± 7)	43.5
Left ventricular ejection fraction (%)	60% (± 5)	55
Previous AF ablations		
0	7 (78%)	2 (100%)
1	2 (22%)	0 (0%)
Pre-procedure medications:		
Amiodarone	6 (67%)	1 (50%)
Beta-blocker	3 (33%)	2 (100%)
Flecainide	0 (0%)	0 (0%)
Digoxin	0 (0%)	0 (0%)
Diltiazem	1 (11%)	2 (100%)
Propafenone	0 (0%)	0 (0%)

## Results

Nine patients with paroxysmal AF were recruited for this part of the study. Table 1 summarises patient demographics for this study, and Table 2 summarises which research protocol was applicable to each patient.

**Table 2:** Summary table of experimental protocols performed

Patient number	Paroxysmal/persistent AF	Threshold assessment	Tau20 Vs Grass for sync HFS-positive GP sites	Tau20 vs Tau20 for sync HFS-positive GP sites	ET-GP mapping and ablation in persistent AF
1	Paroxysmal	✔	✗	✔	✗
2	Paroxysmal	✔	✗	✗	✗
3	Paroxysmal	✔	✗	✗	✗
4	Paroxysmal	✔	✗	✗	✗
5	Paroxysmal	✔	✔	✗	✗
6	Paroxysmal	✗	✔	✗	✗
7	Paroxysmal	✗	✗	✔	✗
8	Paroxysmal	✗	✗	✔	✗
9	Paroxysmal	✗	✗	✔	✗
10	Persistent	✗	✗	✗	✔
11	Persistent	✗	✗	✗	✔

### Threshold assessment

The mean threshold for ET-GP was 34 mA (*n* = 5 sites in 5 patients). The threshold for eliciting a positive response was patient and site specific with a range from 20 to 50 mA. Fifty milliamperes was therefore selected as the current output for subsequent reproducibility assessment to ensure optimal GP detection.

### Reproducibility of GP identification between Tau20 and Grass S88 with SIU5

**Fig. 2:**
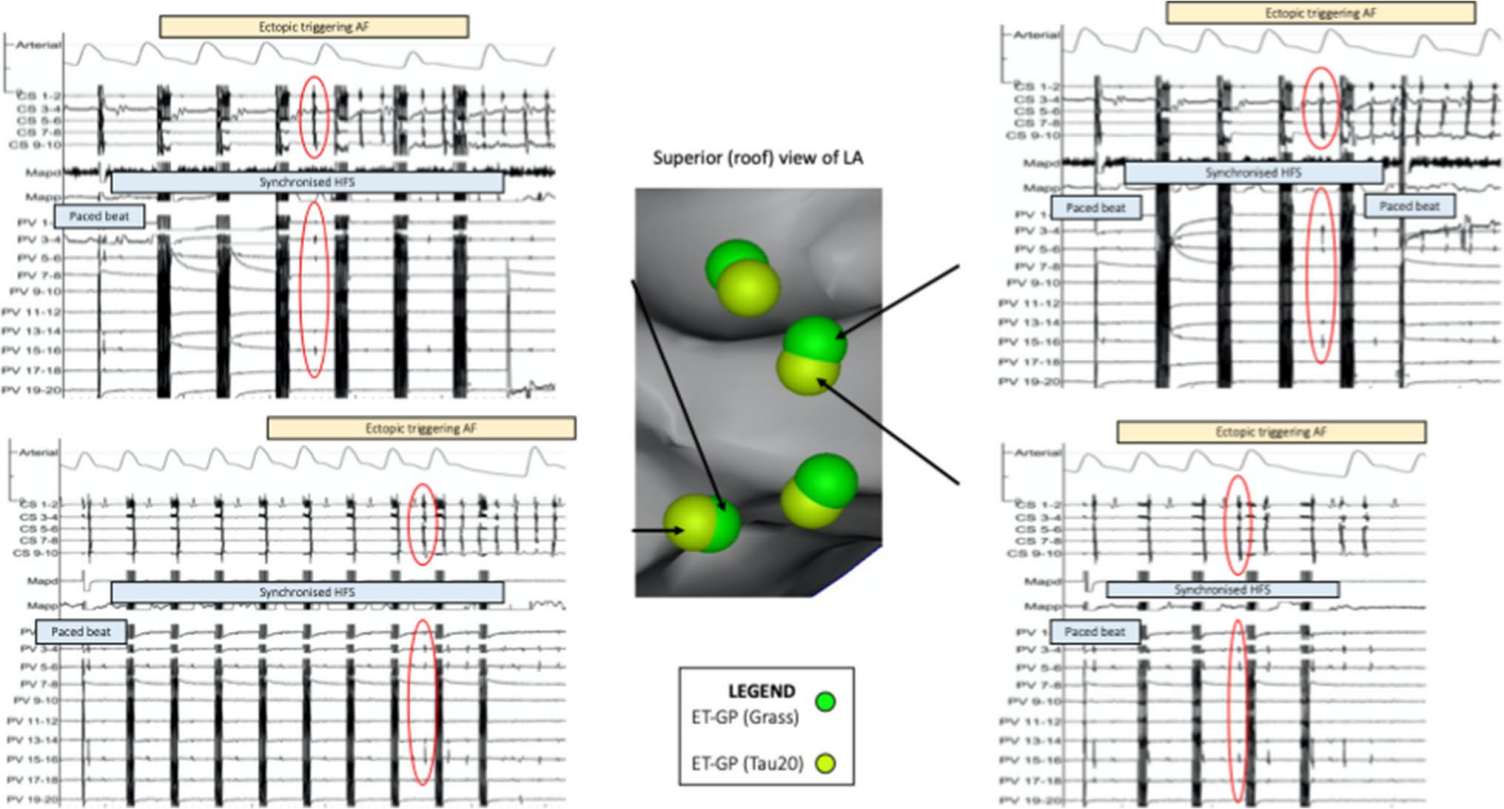
Reproducibility of ET-GP sites identified with synchronised HFS using the Tau20 compared to Grass stimulator. The left atrial anatomy (grey) as visualised with the CARTO™ electroanatomic mapping system. Electrogram (EGM) signals from the EP recording system (LabSystem Pro, Boston Scientific) are shown adjacent. ET-GP sites green. Ectopy circled in red solid lines. HFS applied endocardially via the Map catheter from the Grass stimulator with SIU5 (output 100 V) and then the Tau20 (output 50 mA). Arterial line trace top, CS EGMs, MAP EGMs, PV EGMs bottom

**Fig. 3:**
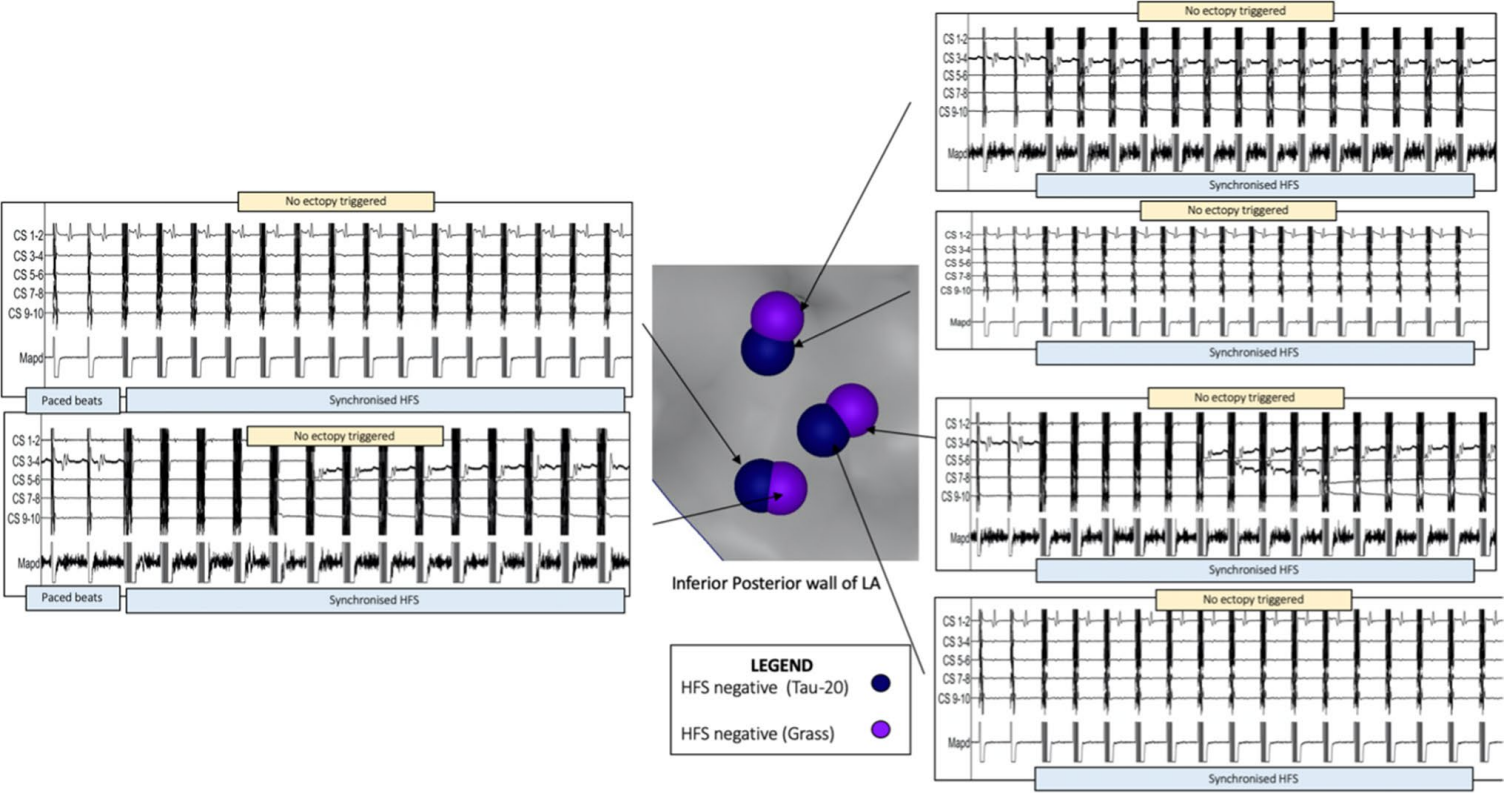
Reproducibility of HFS negative sites with synchronised HFS using the Tau20 compared to Grass stimulator. The left atrial anatomy (grey) as visualised with the CARTO™ electroanatomic mapping system. Electrogram signals from the EP recording system (LabSystem Pro, Boston Scientific) are shown adjacent. Negative synchronised HFS test sites in dark blue/purple. HFS applied endocardially via the Map catheter from the Grass stimulator with SIU5 (output 100 V) and then the Tau20 (output 50 mA). Coronary sinus EGMs above, Map catheter EGMs below

Sixteen synchronised HFS test sites (*n* = 2 patients) were compared between the Tau 20 and Grass stimulator. Reproducibility was 100% (positive, Fig. 2, or negative, Fig. 3) (kappa = 1, SE of kappa = 0.00, 95% CI 1 to 1).

### Reproducibility of GP identification by Tau20

Thirteen synchronised HFS test sites (*n* = 4 patients) were tested multiple times using the Tau20 to ensure repeatable identification of ET-GP sites using the Tau20 stimulator. Reproducibility was 100% (kappa = 1, SE of kappa = 0, 95% CI 1 to 1).

### Feasibility of ET-GP mapping and ablation in persistent AF

Two patients with persistent AF were recruited. Patient 1 had AF lasting 33 months following an initial successful external cardioversion. The left atrial diameter was 4.5 cm with preserved left ventricular function. Patient 2 had persistent AF for 4 months before external cardioversion lasting 2 days. A subsequent cardioversion was performed after 9 months of AF on amiodarone and remained in sinus rhythm until the ablation procedure. The left atrial diameter was 4.2 cm with normal left ventricular function.

**Fig. 4:**
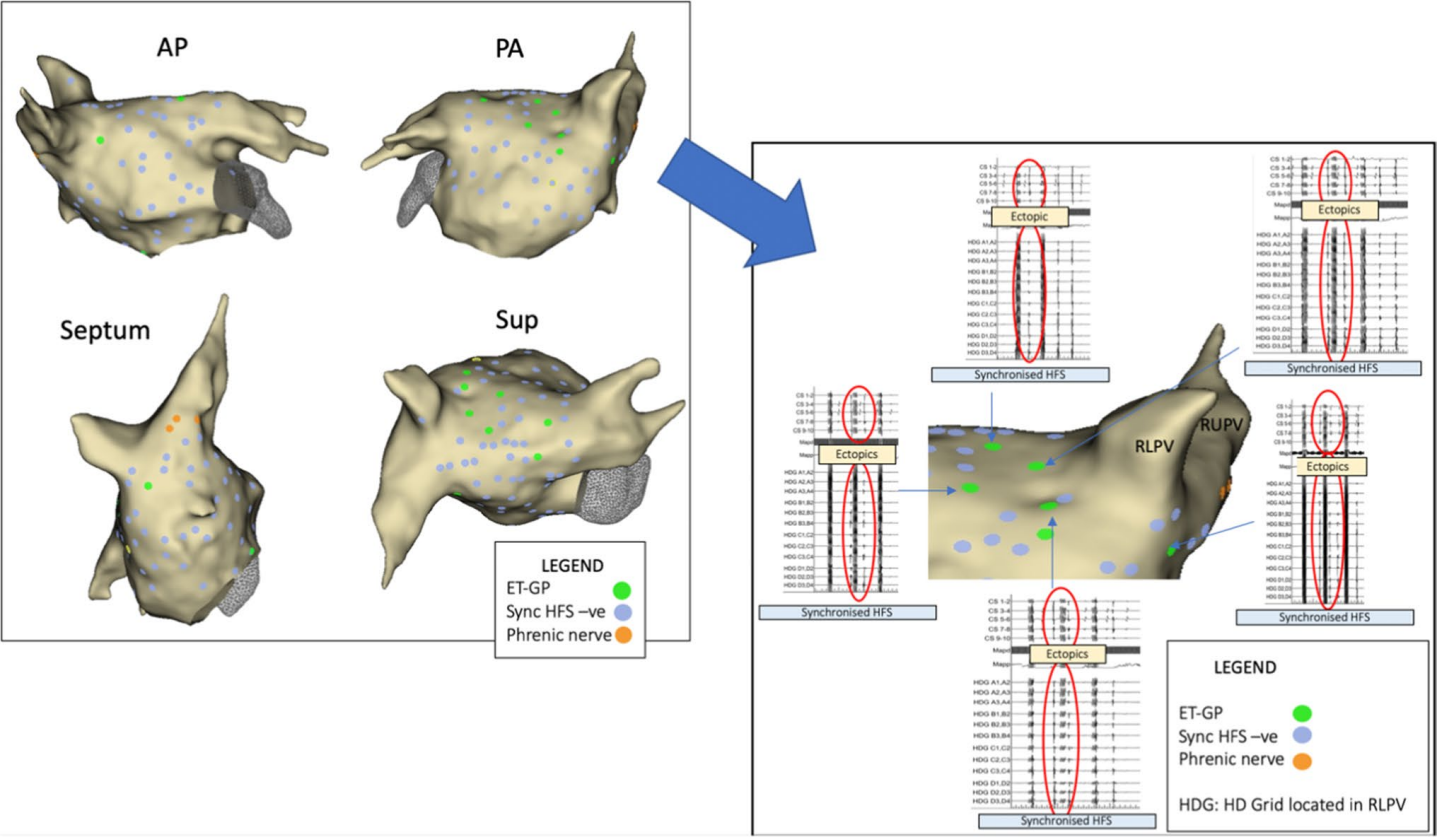
The Tau20 can be used to complete a high-density ET-GP map in a patient with persistent AF in Precision™. Main picture: the left atrial anatomy (grey) as visualised with the Precision™ electroanatomic mapping system. HFS applied endocardially via the Map catheter from the Tau20 (output 50 mA). ET-GP (green), HFS-negative sites (blue), incidental phrenic nerve capture (orange). Second picture: HD grid located in the right lower pulmonary vein (RLPV). Coronary sinus EGMs top, Map catheter middle, HD grid EGMs bottom. Ectopy circled in red solid lines

Patient 1 had 114 sites tested with synchronised HFS from the Tau20 via the Tacticath™ catheter and recorded on the Precision™ system, identifying 10 ET-GP sites (9% of total) (Fig. 4). Each ET-GP was ablated following identification (11 lesions, 9052 J, 6 min 12 s RF duration) and confirmed by negative response to HFS on retesting the site.

**Fig. 5:**
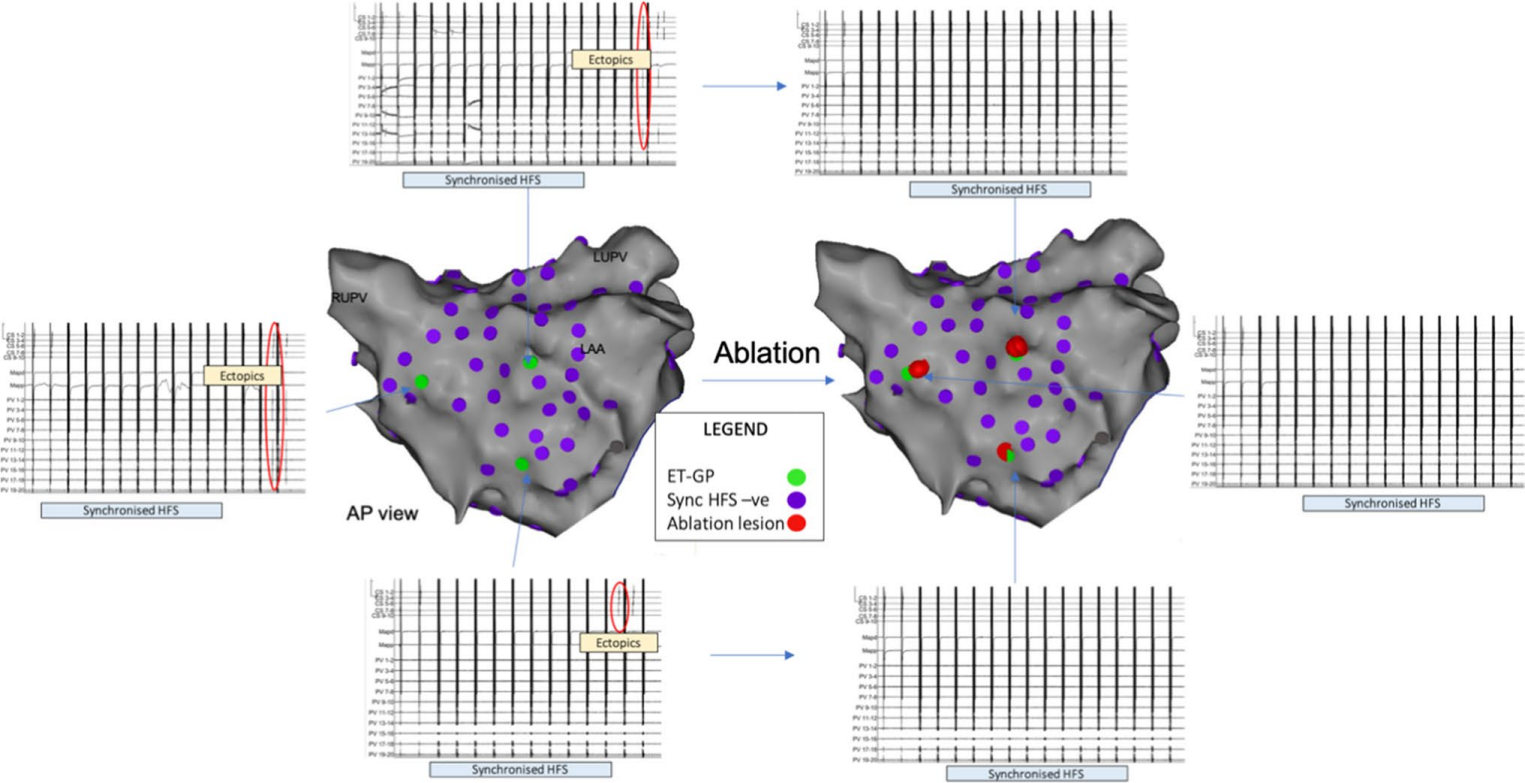
The Tau 20 can be used to identify ET-GP in a patient with persistent AF to guide therapeutic ablation. The left atrial anatomy (grey) as visualised with the Carto™ electroanatomic mapping system. HFS applied endocardially via the Map catheter from the Tau20 (output 50 mA). ET-GP (green), HFS-negative sites (purple). Ablation lesions (red). CS EGMs top, MAP EGMs middle, PV EGMs bottom. Ectopy circled in red solid lines. EGMs left are pre-ablation, and EGMs right are the same sites post ablation confirming efficacy of ablation

Patient 2 had 124 sites tested with synchronised HFS from the Tau20 via the SmartTouch™ catheter and recorded on the CARTO™ system, identifying 7 ET-GP (6%) (Fig. 5). Again, each ET-GP was ablated following identification (7 lesions, 4853 J, 2 min 58 s RF duration) and confirmed with a negative response in retesting.

There were no complications in either case. All antiarrhythmics were stopped after the 3-month blanking period. Both patients underwent regular Holter recordings and ECGs. There was no recurrence of AF following the 12-month review.

## Discussion

This is the first study to confirm that ET-GP can be reproducibly located at the same site by two different stimulators. We used the Tau20 to perform a first-in-man study to show that ET-GP can be identified in patients with persistent AF and provide proof-of-concept that ET-GP ablation alone without pulmonary vein isolation could prevent AF in persistent AF.

The role of the autonomic nervous system in arrhythmia and AF is well known and illustrated in a number of animal studies [6, 14, 15]. The cardiac autonomic nervous system has been assessed as a therapeutic target for AF ablation with mixed results [13, 16, 17]. GANGLIA-AF demonstrated that ablation of GP sites alone can prevent AF recurrence in about 50% of patients with paroxysmal AF [10, 11]. Importantly, this was the first study to specifically target ET-GP rather than AVD-GP. Functional autonomic work in patients with paroxysmal AF has demonstrated that AVD-GP and ET-GP have different anatomical distributions within the atrium [18]. This highlights the importance of functional testing to identify the location of GP and may account for differential success rates in targeted GP ablation. Mapping of ET-GP requires maintenance of SR to enable detection of ectopy. We have demonstrated that it is possible to maintain SR to use synchronised HFS to create a detailed ET-GP map in patients with persistent AF.

It has been suggested that inadvertent ablation of GP during circumferential PV antral ablation may explain the observation of freedom from AF despite PV reconnection [19]. ET-GP are likely to be preferentially ablated compared to AVD-GP during CPVA due to their anatomical distribution, resulting in treatment of the upstream trigger for AF. However, PVI is known to be less successful in patients with persistent AF as compared to paroxysmal AF [20] so non-PV triggers are of greater mechanistic and therapeutic interest. ET-GP are able to trigger PV and atrial body ectopy from sites distant to the PV and outside the line of CPVA. It is therefore important that ET-GP can be selectively mapped and ablated in persistent AF as demonstrated in this proof-of-concept study using the Tau20. The results of this 2 patient pilot study, showing the successful mapping and ablation of ET-GP in patients with persistent AF, with freedom from AF at 1 year, prove that GP mapping is not only feasible in this cohort of patients, but also worthy of significant further investigation.

Ablation of targets in addition to PVI is common in the treatment of persistent AF. This can lead to significant amounts of RF ablation in atria that often already have a high burden of scar [21]. The ability to map GP, tailored to the ET-GP sub-group, in persistent AF enabled the two patients presented here to have very limited ablation. A larger study is required to see if this limited ablation in persistent AF can achieve similar outcomes to the GANGLIA-AF study which achieved freedom from AF with up to 56% the amount of RF energy used in conventional PVI.

PV isolation has been the cornerstone of AF ablation since the early 2000s but despite significant advances in techniques to enable durable PVI, further improvement in outcomes is needed [22, 23]. That GP ablation (without PVI) can prevent AF recurrence [11] which implies GP sites are part of the AF mechanism even in persistent AF and so should be investigated further as a potential adjunct to improve outcomes. This could be in combination with or post PVI (at a first time or a redo procedure respectively). To minimise additional procedure time, high-density GP mapping could be limited to identification of non-PV triggers outside the PV antrum or combined with single-shot PVI technology.

## Limitations

We compared only two stimulators and we do not know if this reproducibility extends to other commercially available stimulators. The Grass stimulator has been the most widely used for autonomic studies and the Tau20 was designed to have a similar performance whilst meeting current regulatory standards. However, should similar parameters be used in further studies, then similar results would be expected.

A further limitation is the small sample size of this study. Whilst the size is adequate to ensure that the Tau20 performs consistently and to an equal or better standard than the Grass stimulator, further studies into the efficacy of mapping and ablation of ET-GP in persistent AF are warranted before any definitive conclusions can be drawn. This study does provide compelling evidence that a further study into this field is warranted.

## Conclusion

ET-GP sites can be located with a high degree of reproducibility by different stimulators. ET-GP sites can be identified in persistent AF and ablation of ET-GP can prevent recurrence of persistent AF.
